# Adolescent Connectedness: Testing Confirmatory Factor Analysis of the Hemingway: Measure of Adolescent Connectedness–Bahasa Melayu Version (HMAC–BM)

**DOI:** 10.3390/ijerph191912189

**Published:** 2022-09-26

**Authors:** Nor Azzatunnisak Mohd Khatib, AbRahman Roseliza-Murni, Suzana Mohd Hoesni, Jamiah Manap

**Affiliations:** Center for Research in Psychology and Human Well-Being, Faculty of Social Sciences and Humanities, Universiti Kebangsaan Malaysia, Bangi 43600, Malaysia

**Keywords:** adolescents, connectedness, confirmatory factor analysis, HMAC‒BM

## Abstract

Measuring the factors that underlie adolescent connectedness has become a prominent focused issue in past studies across many disciplines. Thus far, the Hemingway: Measure of Adolescent Connectedness (HMAC) is the first research-based measure of adolescent’s relationship and sense of belonging with other people and their surroundings. The current study aimed to examine the measurement model of the Hemingway: Measure of Adolescent Connectedness which has been translated into Bahasa Melayu (HMAC–BM) in order to check for its feasibility among Malaysian adolescents. A total of 377 adolescents aged 16 years old were recruited from the Federal Territory of Kuala Lumpur. Three factors, namely connectedness to family, school, and neighbors with seven sub-factors of the HMAC–BM, were analyzed by Confirmatory Factor Analysis (CFA) using the IBM SPSS Amos 23.0 (23.0, IBM Technology, Armonk, NY, USA). Results of the CFA supported the second-order factor of the HMAC–BM structures. The overall HMAC–BM scale and its subscales have higher factor loadings ranging from 0.60 to 0.79. Cronbach’s alpha coefficients ranged from 0.78 to 0.95 for the three subscales and 0.84 for the total scale. Results also revealed seven sub-factors with forty-one factors—solution that accounted for 0.89% of total variance explained for adolescent connectedness. Findings provide empirical support for the feasibility of HMAC–BM in explaining Malaysian adolescents’ social connectedness. Hence, the HMAC–BM is a promising measure that can be used on Malaysian adolescents. The findings have important implications which provide a clear picture of HMAC–BM as an accurate instrument to measure adolescent’s social connectedness toward enhancing prosocial attitudes and well-being.

## 1. Introduction

Adolescence is a time of great transitions as one leaves childhood behind. It is a period of development that marks the beginning of adulthood and personal independence. Filled with rapid social, psychological, and physical changes, adolescence is indeed a period of enormous changes and challenges. In fact, during adolescence, crucial developmental events such as identity development, attachment, social belonging, and close relationship development start to occur [1]. Adolescents start to reconnect with friends, family members, and social communities to explore their place in the world. At this time, connectedness plays an important protective factor for the adolescent to reduce the likelihood of a variety of risky behaviors [2]. Adolescent connectedness is a term that is widely used in the previous literatures [2,3] to explain the complex social network of one’s peers and family, who provide the building blocks for setting goals and aspirations. In previous works, adolescents’ connectedness is synonymous with several other terms such as belonging, closeness, social cohesion, and social connection. The terms adolescent’s connectedness and social connectedness were used interchangeably and defined in a myriad of ways [2,3]. Social connectedness also defined as the subjective awareness of being in close relationships with the social [4]. In contrary, social connectedness also related to digital practices [5] which from the technological perspective, connectedness refers to communication tools or the use of technology as a medium to enhance social connectedness and well-being [6]. These definitions have grown out of the literature about adolescent connectedness. It can more clearly be seen based on affective and behavioral aspects in a relationship and also activities that are consistent with the general view of connectedness perspectives [7].

In early adolescence, social connectedness helps in social-emotional development and also verifies their optimal mental and physical development [8]. At this age, adolescence offers both opportunities and risks for maintaining a sense of connectedness [9]. Nevertheless, in maintaining a positive effect on adolescent well-being, adolescent connectedness becomes a key component for positive youth development which are associated with various positive life outcomes [10]. Thus, a variety of research on adolescents’ connectedness was conducted in many disciplines related to health [6,7], education [8,10] and also those related to ethnic identity [11]. In social sciences, many researchers, for instance, are more interested in studying adolescent connectedness in a school system ecology that targets students’ and teachers’ relationships [8,12]. Meanwhile, family connectedness focuses more on parents, siblings, and family members [11,13]. Whereas, neighbor connectedness focused on neighborhood social ties which depend on location but not relationships that one necessarily seeks or that are familial in nature [14]. Within the perspective of criminals, connectedness is merely focused on community connectedness among young male prisoners [15]. Whereas, in other contexts, the social connectedness was also conducted involving middle-aged working women with career satisfaction, engagement, and social support on their life satisfaction [16].

Previous studies revealed that connectedness in adolescents is associated with various positive outcomes [10]. Hence, research on the adolescents’ connectedness has been conducted in different settings and disciplines. Studies on the role of connectedness in the development of children and adolescents are also accumulating rapidly [7]. The present study uses the Bronfenbrenner Ecology System Theory to explain the relationship of each dimension of connectedness, namely family, school, and community connectedness in adolescent’s perspectives. According to [1,17], positive social connectedness increases the chances of adolescents’ optimism and life satisfaction. Each of the connectedness plays its own role in empowering adolescent capability to adapt with their environment. A harmonious and constructive school environment, for example, can induce happiness to adolescents which indirectly affects their academic achievements [18] and well-being [17]. Adolescent connectedness impact, based on a previous study [18], can clearly be seen in terms of emotional stability and their prosocial behavior. This considered, positive adolescent connectedness would strengthen healthy relationships with others. Results from the previous study [19] elucidates that peer relationships at school can improve the efficiency of school connectedness and a sense of belonging. While, a study by Kiely et al. [20] showed a significant relationship between school connectedness and prosocial behavior. Results from the previous research [21] also showed that school connectedness has a significant relationship with family connectedness and self-mastery. School connectedness and self-mastery prevent teenagers from being involved with negative influences that weaken family functionality. Likewise, neighbor’s connectedness is important to form the most perfect community network [22], which becomes a determinant for well-being through the cooperation and unity that was formed. However, the influence of the residential area or neighborhood is usually not considered and is less studied [23] which needs further clarification.

Previous studies are uniformly consistent in documenting the adolescents’ connectedness as a positive factor of happiness and psychological well-being [14,16]. The degree of adolescent connectedness to different social domains (i.e., family, school, and neighbors) have also been shown to give an impact not only on their positive development and lifestyle, but also to improve their sense of well-being [23]. Multiple studies were conducted separately and focused on certain connectedness such as school, friends, neighbors, community, teachers, and family. Thus far, only few studies have examined adolescent connectedness involving multiple perspectives of adolescents’ connectedness, namely connectedness to family, school, and neighbors simultaneously [10], compared to specific adolescence connectedness to school [2], parents [3], and community [24], which were conducted separately. Adolescent social connectedness has been extensively covered in previous research and was used on numerous scales to examine the different perspectives of adolescent connectedness in a school setting [25,26], community [27], family [28], and combining multi-perspectives of connectedness [29]. One of the measures which was commonly used in previous studies to measure adolescent connectedness that implicates many perspectives of connectedness was the Hemingway: Measure of Adolescent Connectedness (HMAC). It is the first research-based measure of an adolescent’s relationship and sense of belonging with other people.

In early years, HMAC was introduced to measure adolescent connectedness only in school settings. Nowadays, it has been extended to other disciplines to measure a wide range of adolescents’ connectedness [23,25]. The HMAC psychometric properties have been tested in Taiwan [30], Chile [31], and the United States of America [32]. Findings from previous studies reported that HMAC is a promising instrument to measure adolescent connectedness. Different results were revealed through the validation process using Exploratory Factor Analysis (EFA) [30,32] and measurement invariance testing across gender and ethnicity [33]. The EFA test using HMAC conducted with a Chilean sample showed an 11-factor solution [31]. The 11-factor solution with 57 items explained 61.92 percent of the variance of all factor coefficients which ranged from 0.32 to higher. There were no cross-loaded items found in the HMAC and it became a reliable instrument to measure adolescents’ connectedness across numerous domains of Chilean, Taiwanese, and South African samples. Likewise, the measurement invariance across gender, racial, and ethnic groups of adolescent connectedness measures also provide concrete evidences of factorial validity evidences [33].

On the other hand, there were also past studies which revealed the limitation of the data in which some of the adolescents’ connectedness research showed inconsistencies in the findings, which suggests the need for additional psychometric studies to certify the applicability of the measures [22]. In addition, to clarify the lack of adolescent connectedness studies, [25,26] suggested the need for research in connectedness to be tested by combining multiple dimensions of connectedness and providing substantial conceptual clarification. Previous research also [10] found that no study has summarized data on the existing measures of adolescent connectedness. Another issue being identified with using multi-dimensional measures is that the operationalization of many concepts often overlaps and is not easily distinguishable from one another [8]. The gaps from previous studies [9,34] prompt the present study to be conducted to examine multiple dimensions of adolescents’ connectedness that implicates, namely school connectedness, family connectedness, and neighbor connectedness among Malaysian adolescents and, subsequently, testing the translated Hemingway Measure of Adolescent Connectedness–Bahasa Melayu version (HMAC–BM).

### Present Study

Specifically, the present study had two main objectives. First, we aimed to analyze the descriptive profile of adolescent connectedness involving gender, race, and household income of the participants. Next, we tested the feasibility of the HMAC–BM measure among Malaysian adolescents, implicating measuring family connectedness, school connectedness, and neighborhood connectedness using second-order factor CFA models. We hypothesized that three main dimensions of adolescent connectedness (family, school, and neighbors) with the seven sub-factors tested using confirmatory factor analysis CFA. We also assumed the measurement model would fit to the current data and all sub-factors would contribute to explain the adolescents’ connectedness in the capital city of Malaysia. Measurement models were analyzed using IBM SPSS Amos 23.0 software (23.0, IBM Technology, Armonk, NY, USA). To date, the adolescent is highlighted in an urban area in the Federal Territory of Kuala Lumpur or the capital city of Malaysia and focused on national secondary school. We expected that the seven sub factors of the HMAC–BM measure would consistently fulfill the threshold required and be well-fitted with the current data used.

## 2. Materials and Methods

### 2.1. Participants

The participants were 377 adolescents (n = 219 male, n = 158 female) of whom were national secondary school students selected from the three zones in the Federal Territory of Kuala Lumpur. The participant was 16 years of age and was selected using stratified random sampling. Data were gathered from the survey involving self-administered questionnaires using paper-pencil surveys to obtain quantitative data.

### 2.2. Measures

The Hemingway: Measure of Adolescent Connectedness (HMAC) was used to measure adolescent connectedness to family, school, and neighbor. The measure has been translated and validated from English into the Bahasa Melayu version using the 5 steps of back-translation method and bilingual technique proposed by Jones (2001) [35]. First, an informed and an uninformed translator independently forward-translated the items from English to Bahasa Melayu. In the second step, the target language version was blindly translated by the blind translator. In the third step, the four bilingual people had a group discussion to identify any differences between the target and source versions; who resolved incongruities between the translations and produced the translation. In the fourth step, the new version was independently back-translated by two more bilingual people. In a fifth step, the forward- and back-translations were examined by a bilingual committee encompassing all the above-mentioned translators and then the translated version. Lastly, the reliability and equivalence were tested on a sample of 125 participants and reported in pilot study.

#### 2.2.1. The Hemingway: Measure of Adolescent Connectedness

To measure the facet of adolescent connectedness, we used the Hemingway: Measure of Adolescent Connectedness (HMAC) version 5.5 developed by [32]. In the present study, the self-report survey consisted of 36 items that were used to measure adolescents’ degree of involvement in specific relationships, contexts, and activities. The participants were asked to rate each item using a 5-point scale (1 = not at all true) to (5 = very true). The negative item responses (2, 6, 14, 19, 24, 32, 37) were reverse coded. Seven factors focus on (1) neighbor connectedness; (2) friend connectedness; (3) parent connectedness; (4) sibling connectedness; (5) school connectedness; (6) peer connectedness; (7) teacher connectedness. Item score for each scale was averaged to a composite score which covers three composites of adolescent connectedness, namely: (i) family connectedness (parent connectedness and sibling connectedness); (ii) community connectedness (friend connectedness and neighborhood connectedness); (iii) school connectedness (school connectedness, peer connectedness, and teacher connectedness). Several changes were made from the original version after an exploratory factor analysis (EFA) was conducted. The original version of the measure consisted of self-connectedness, but in the present study, it was omitted to avoid overlapping with the self-compassion. Overall score was computed as a grand mean of all items. The results of the internal consistency coefficient were found as α = 0.84 in the present study.

#### 2.2.2. Socio-Demographics

We requested demographic details from the participants consisting of gender, race, and household income.

### 2.3. Procedure and Statistical Analysis

Ethical approval was granted by the Educational Planning and Policy Research Division (EPRD) under the Ministry of Education which is the unit responsible for educational planning, research evaluation, policy analysis, and coordination. Data were collected in 2019 via a paper-pencil survey. Study eligibility was limited to citizens of Malaysia who were secondary schooling students aged 16 years and fluent in Bahasa Melayu language. Parental informed consent was obtained as the participants were considered under-aged. After the parental permission was granted, the participant filled up their own written informed consent on the day data collection was held. Participants were encouraged to answer all the questions and were free to omit items they did not wish to respond to, but were prompted to attend to missing data entries. At the conclusion of the survey, participants received written debriefing information and were remunerated MYR 3 as a token of appreciation for their participation. After all the questionnaires were gathered, data were treated by checking the missing responses in a dataset and further analyses were conducted using SPSS-AMOS 22 to test the measurement model of the Hemingway: Measure of Adolescent Connectedness–Bahasa Melayu Version of (HMAC–BM).

## 3. Results

### 3.1. Descriptive Informations

Table 1 presents the descriptive statistic. Out of the 377 adolescents that were included in the analysis, 58.1% (n = 219) were male and 41.9% (158) were female. The imbalance of male and female participants resulted from the selection process which referred to the school capacity of the students. Respondents were 16 years old. Majority of the respondents were Malays 64.5%, followed by Chinese were 24.1%, Indians were 10.1%, and the remaining balance was from other races 1.3%. The household income was divided into four categories. The findings of the study showed the total household income of the respondents’ families with incomes less than MYR 1000 were 14.3%, incomes from MYR 1001 to MYR 3000 were 57.8%, incomes from MYR 3001 to MYR 5000 were 19.9%, and incomes more than MYR 5000 were 7.4%, and 0.5% of the respondents chose not to answer. Most of the respondent’s family incomes fell under the second category (MYR 1001-MYR 3000), which is 57.8%.

#### Adolescent Connectedness

Table 2 presents the mean scores and standard deviation of all factors and sub-factors of adolescent connectedness. The mean score for total adolescent connectedness showed the value of 142.34 (±23.20). While the mean and standard deviation for seven sub-factors of adolescent connectedness showed neighbor connectedness at 21.25 (±4.91), friend connectedness at 21.55 (±4.89), parent connectedness at 23.78 (±4.44), sibling connectedness at 14.63 (±3.35), school connectedness at 23.11 (±4.62), peer connectedness at 21.29 (±5.33), and teacher connectedness at 16.70 (±3.71). Adolescents reported spending the most of their time engaging with parents. The result showed that parent connectedness was among the highest mean score at 23.78, compared to the lowest mean score of sibling connectedness at 14.63. The results of the descriptive analysis showed the adolescents spent more time with their parents compared to other connectedness groups. They value time with their parents more compared to the other connectedness groups, and the results are reported in Table 2.

### 3.2. Confirmatory Factor Analysis

**Figure 1:**
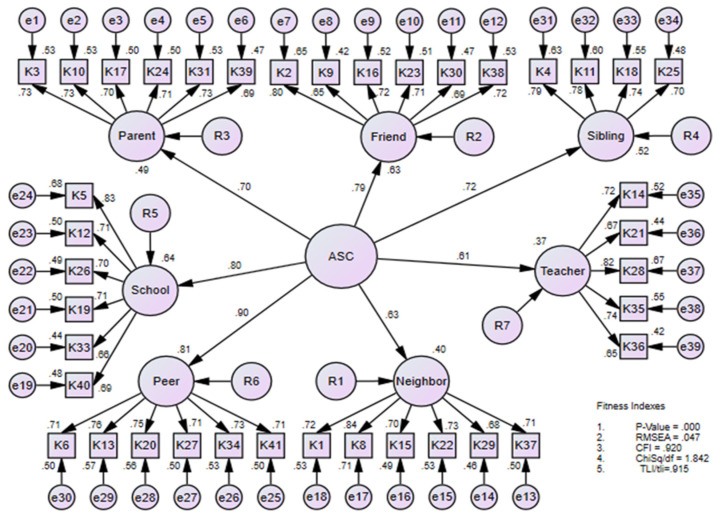
Second-order 7-factor confirmatory factor analysis model (n = 377). Note. RMSEA: Root Mean Square Error of Approximation; CFI: Comparative Fit Index; TLI: Tucker–Lewis Index. ASC = adolescent social connectedness; parent = parent connectedness; school = school connectedness; Peer = peer connectedness; Neighbor = neighborhood connectedness; Teacher = teacher connectedness; Sibling = sibling connectedness.

A measurement model of adolescent connectedness was tested using the second-order confirmatory factor analysis (CFA) using second-order seven factors of HMAC–BM. In the model (Figure 1), the formative factors of adolescent social connectedness were labeled using the acronym, namely ASC = adolescent social connectedness; parent = parent connectedness; school = school connectedness; Peer = peer connectedness; Neighbor = neighbor’s connectedness; Teacher = teacher’s connectedness; Sibling = sibling’s connectedness. The acronym of K represents the observed score for adolescents’ social connectedness. The acronym of e = error in the equation or measurement for items and R1 to R7 = residual. Results of confirmatory factor analysis have confirmed that seven factors represented the adolescent connectedness. The factors emerged after the exploratory factor analysis was performed. Then, the CFA was conducted using a theoretical assumption that matches the reality which is the actual data presented. Factor loading for each item for this model is demonstrated in (Figure 1). We used the root mean square of error approximation (RMSEA) and the result showed that RMSEA = 0.05 ranged from 0.03 to 0.08 achieving the threshold. While Comparative Fit Index (CFI) values of 0.92 exceeded 0.90 indicative of adequate fit, Tucker–Lewis Index (TLI) as statistics determine model fit showed 0.92, which exceeded 0.90 indicative of adequate fit and CMIN/DF = 1.84 less than 5.00, also indicative of good fit achieving the threshold required. Additional steps were used to further assess the goodness-of-fit using the normed model chi-square (χ^2^/df; values < 3.0) considered as good. The Chi-Square Goodness-of-fit test for social connectedness was significant [(χ^2^ (N = 377, DF = 695) = 1280.43, k < 0.05)] at (*p* < 0.001). The result showed that all items achieved the factor loading threshold and fit the data sufficiently (see Figure 1). The standardized latent factor loadings of 41 items ranged from 0.60 to 0.82. Most of the items in adolescents’ connectedness sub-factors had factor loadings ranging from 0.60 to higher, which is considered an acceptable cut-off value. The results of the second-order confirmatory factor analysis (CFA) for adolescent connectedness displayed adequate construct validity after achieving the prerequisite for all three fitness indices.

Table 3 presented adolescent connectedness with seven correlated factors that achieved the threshold and fit the data sufficiently. The results of the overall analysis are shown in (Figure 1) and the factor loading for each showed the RMSEA, CFI, TLI, and CMIN/DF achieved the threshold and the detailed explanation of the results is in (Table 4).

The average variance extracted (AVE) for adolescent connectedness achieved the convergence validity which the value of AVE = 0.52 exceeded the standardized factor loading estimates higher than 0.50, ideally. Similarly, composite reliability (CR) = 0.89 indicated less than the required threshold which is 0.60 and above. Each latent construct for the adolescent connectedness showed peer connectedness = 0.52, parent connectedness = 0.51, school connectedness = 0.51, peer connectedness = 0.53, neighbor connectedness = 0.53, sibling connectedness = 0.58, and teacher connectedness = 0.52 showed the value exceeded the threshold. The confirmatory factor analysis (CFA) analysis showed that convergence validity and composite reliability for adolescent connectedness were achieved and fit the data sufficiently. All the latent constructs of the adolescent connectedness were statistically identified. The confirmatory factor analysis of HMAC–BM was conducted as a perquisite to proceed with a further analysis which is structural equation modeling (SEM). The result of the present study enables the HMAC–BM to be included in the structural equation model for further analysis. The details of AVE and CR values for the adolescent connectedness are stated in (Table 4).

The present study has revealed that the adolescent connectedness items are eligible to be included in the structural equation model analysis and proceeded for the advanced analysis. The details of AVE and CR values for each dimension of the adolescents’ connectedness are shown in (Table 5).

## 4. Discussion

Adolescent connectedness to broad perspectives of connectedness seems to be crucial to adolescent’s development to prevent mental health problems [36], juvenile delinquency [37], and bullying [38]. The understanding about the functional adolescent connectedness leads to a new pathway toward creating positive adolescent development [39] and happiness [40]. However, the majority of the previous research does not cover multiple connectedness simultaneously [34], which means the exact component of adolescent connectedness remains scarce. In accordance with the previous gap, the present study investigated the measurement model of adolescent connectedness of the HMAC–BM measure in the Malaysia perspective. Assuming that adolescent’s connectedness has limited resources to explain inclusive perspectives of general connectedness among adolescents, we tested whether the HMAC–BM measure might be an adequate scale to measure adolescents’ connectedness. The findings of the present study support the conclusion drawn from prior research [30,41] that showed the overall HMAC–BM and the subscales of family connectedness (parents and siblings), school connectedness (school environment, teachers, and peers), and neighborhood connectedness (friends and neighbors) are congruent with theoretical expectations [25,26] that were introduced by [32]. The hypothesized second-order seven-factor models were found to be the best fit model to the data, suggesting that all seven factors have distinctive contributions to the latent construct. The findings of the present study imply that a seven-factor structure of adolescent connectedness has been confirmed in several studies that was conducted separately on specific connectedness in an example of school connectedness [32]. The subscales of school connectedness had low factor loadings compared to another connectedness in the HMAC–BM version. The value considered as a good and acceptable cut-off within the accepted threshold is more than 0.60 compared to the Chilean sample [42].

This study provided evidence concerning the general adolescent connectedness model which implicates three main connectedness include family, friend and neighbor connectedness similar to the previous findings [34]. Results showed that the model proposed was well-fitted with the current data. The results also indicated that adolescents are more connected to siblings rather than parents in the family. Family connectedness seems to be higher than other groups of connectedness. The results portrayed that families are higher among adolescents, consistent with prior research [43], the adolescent spent more time with their family compared to friends, and school connectedness can promote resilience [43]. Yet, this current study’s findings are also parallel with the previous study, which revealed the role of family connectedness in explaining the meaning of life [11,44]. This considered, the relationship between parent and child triggers children to appreciate their father’s love and make it a reason to be successful in life [45]. These associations were significant even after accounting for race, gender, and household income. Past research has explained that family connections and closeness to parents seem to a have significant influence on children’s happiness [40]. Despite demonstrating that family connectedness is higher compared to other groups of connectedness, this study also demonstrates that high connectedness in family is also contributed by the positive association among the siblings which partially support the strong family ties to become stronger. This study reported the strongest evidence on convergent validity of family connectedness, which supports the prior research, suggesting that family connectedness predicts some positive adolescent life outcomes better than other types of connectedness [22].

The present study also supported the results of [32] that revealed adolescents’ connectedness measurement models were fitted in the current data in explaining three composite constructs for family, school, and community perspectives. The findings suggest that the model of adolescent social connectedness may provide a useful lens through which to view adolescent connectedness and helps in examining the psychosocial development process of adolescents [37]. The important limitation of this study was that the data were weighted to approximate representativeness of Malaysian adolescents so the findings may not represent all Malaysian adolescents due to a selection of the sample size that only focused on the area of the Federal Territory of Kuala Lumpur and its response rate. Future studies are suggested to add more samples that can represent the whole region of Malaysia in explaining adolescents’ connectedness in a broader perspective.

The current study also showed the other perspectives of adolescent connectedness whereby, school connectedness turns out to be the other prominent factor that can contribute to adolescents’ well-being, next to family. Generally, the current study showed that each type of connectedness plays its own role to enhance adolescents’ psychosocial competencies [37] and their well-being [46]. Our results are consistent with the previous studies [14,29], showing that family connectedness and school environment plays an important role in an adolescent’s life. Although this study found disparities in explaining each of the adolescents’ connectedness, we found that the hypothesized model of adolescents’ connectedness can reduces all these disparities. The present study supported the prior research mentioned; in the school microsystem of connectedness to others, the adolescents were highly connected to their parents (especially to their mothers) and to their teachers at school [47]. Theories of ecological systems also have explained that the adolescents who get connected with general connectedness build a positive surrounding, promote good internal assets among at risk youth [48] and increase psychological well-being. Nevertheless, the findings in the present study must be interpreted by considering the following limitations. First, the study sample was restricted to adolescents who were from the capital cities of Malaysia, Kuala Lumpur. It was a cross-sectional design and conclusions about the cause-and-effect relationship between social connectedness and well-being, as well as the effect of family, school, and community connectedness, cannot be made to generalize about the connectedness of the Malaysian adolescent population.

Given the limited number, participants also involved the secondary school age of 16 years which is the age of middle adolescent in the developmental perspective. Looking at unaccompanied data from other studies involves different level of age in Malaysia, the limited scope of the current study has become evident for future research should examine this. The HMAC–BM measure does not consider the characteristics or quality of the relationships or adolescent connectedness in detail. Despite the limitations, the present study has implications for further refinement of the HMAC–BM measure. The proposed model has a potential for helping the practitioners to understand the mechanisms by which adolescents become connected to myriad connectedness platforms that give the positive outcomes from the connection they have. It also could help in developing a comprehensive adolescent connectedness model to increase the functioning of each connectedness particularly among vulnerable adolescents in a collectivism culture. On behalf of items in the survey design, it should be noted that all HMAC–BM items were mixed with positive and negative items; therefore, either modifying them into negative or positive statements or mixing them with other negative items could lead to distortion of the results. Given that the variety of identifying types of item responses can possibly result in different results, future research should also identify strategies to apply the HMAC–BM for adolescents and mitigate their biases and misunderstandings.

## 5. Conclusions

Adolescents who experience a wide range of positive connectedness increase their subjective well-being, prevent delinquency and criminal behavior, and offer a good developmental outcome. Results of this study provide empirical support about the adolescent connectedness closeness to family, friend, school, and neighbor. By knowing adolescent connectedness, we can understand the proper strategies to enhance and promote prosocial behavior and support their positive psychosocial development process. The result is that compromising demonstrated adolescent connectedness can bring enormous outcomes in an adolescent’s life. It is recommended to use this study outcome, especially when the aims are apt to increase or strengthen adolescent potential toward positive adolescent development. The biggest limitation of the present study was the demography which only focused on one state, that is the capital city of Malaysia. It is recommended to replicate the study into a bigger scope of representative samples. Further studies suggested investigating each domain of the adolescent’s social connectedness using more samples and focusing across the states of Malaysia or worldwide population, which is necessary to specifically address possibilities related to adolescent connectedness.

## Figures and Tables

**Table 1:** Descriptive statistic (n = 377).

Demography	Frequencies	Percentage (%)
Zone		
Sentul	186	49.3
Keramat	123	32.6
Bangsar/Pudu	68	18.0
Gender		
Male	219	58.1
Female	158	41.9
Race		
Malay	243	64.5
Chinese	91	24.1
Indian	38	10.1
Others	5	1.3
Household income		
<1000	54	14.3
MYR 1001–MYR 3000	218	57.8
MYR 3001–MYR 5000	75	19.9
>MYR 5000	28	7.4

**Table 2:** Descriptive information on adolescent connectedness.

Construct and Dimensions	Mean
Adolescents’ Social Connectedness	142.34 (±23.20)
Neighbor Connectedness	21.25 (±4.91)
Friend Connectedness	21.55 (±4.89)
Parent Connectedness	23.78 (±4.44)
Sibling Connectedness	14.63 (±3.35)
School Connectedness	23.11 (±4.62)
Peer Connectedness	21.29 (±5.33)
Teacher Connectedness	16.70 (±3.71)

Note: M (SD) = mean (± standard deviation) for adolescent connectedness.

**Table 3:** Fitness indices of adolescent connectedness.

Index	Required Fitness Index	Hypothesize Model
*p*-value	>0.05	0.00
RMSEA	0.03–0.08	0.05
CFI	>0.90	0.92
TLI	>0.90	0.92
CMIN/DF	<5.00	1.84

Note. RMSEA: Root Mean Square Error of Approximation; CFI: Comparative Fit Index; TLI: Tucker–Lewis Index; CMIN/DF: minimum discrepancy per degree of freedom.

**Table 4:** Average Variance Extracted (AVE) and Composite Reliability (CR).

Factor	Sub-Factors	Factor Loading	CR (>0.6)	AVE (>0.5)
Adolescent connectedness	Friend connectedness	0.79	0.89	0.55
	Parent connectedness	0.70		
	School connectedness	0.79		
	Peer connectedness	0.90		
	Neighbor connectedness	0.63		
	Sibling connectedness	0.72		
	Teacher connectedness	0.61		

**Table 5:** Average Variance Extracted, Composite Reliability, and Factor Loadings for adolescent measurement model (n = 377).

Factors	Items	Loading Factor	CR (>0.6)	AVE (>0.5)
Friend connectedness	K2	0.81	0.86	0.52
	K9	0.65		
	K16	0.72		
	K23	0.71		
	K30	0.69		
	K38	0.73		
Parent connectedness	K3	0.73	0.86	0.51
	K10	0.73		
	K17	0.71		
	K24	0.71		
	K31	0.73		
	K39	0.69		
School connectedness	K40	0.69	0.86	0.51
	K33	0.66		
	K19	0.71		
	K26	0.70		
	K12	0.71		
	K5	0.83		
Peer connectedness	K41	0.71	0.87	0.53
	K34	0.73		
	K27	0.71		
	K20	0.75		
	K13	0.76		
	K6	0.71		
Neighbor connectedness	K37	0.71	0.87	0.53
	K29	0.68		
	K22	0.73		
	K15	0.70		
	K8	0.84		
	K1	0.73		
Sibling connectedness	K4	0.79	0.87	0.58
	K11	0.78		
	K18	0.74		
	K25	0.70		
	K4	0.79		
Teacher connectedness	K14	0.72	0.84	0.52
	K21	0.67		
	K28	0.82		
	K35	0.74		
	K36	0.65		

Note: K = item of the factors in the Hemingway: Measure of Adolescent Connectedness.

## Data Availability

The data is available upon request from the authors.
